# Pattern of cognitive impairment among community-dwelling elderly in Egypt and its relation to socioeconomic status

**DOI:** 10.1186/s42506-023-00147-3

**Published:** 2024-02-08

**Authors:** Amany A. Tawfik, Sarah A. Hamza, Nermien N. Adly, Radwa M. Abdel Kader

**Affiliations:** 1 Department of Geriatrics and Gerontology, Faculty of Medicine, Helwan University, Cairo, Egypt; 2 Department of Geriatrics and Gerontology, Faculty of Medicine, Ain Shams University, Cairo, Egypt

**Keywords:** Ain Shams Cognitive Assessment tool, MoCA, Pattern of cognitive impairment, Socioeconomic status, Community-dwelling elderly

## Abstract

**Background:**

Cognitive decline is one of the aging health problems that strongly affects daily functioning and quality of life of older adults and threatens their independence. The aim of this study was to assess the prevalence and pattern of cognitive impairment (CI) among community-dwelling elderly in Egypt and the contribution of socioeconomic status to inequality in cognitive impairment.

**Methods:**

A cross-sectional study involved 470 community-dwelling elderly aged 60 years or older living in Kafr El-Sheikh Governorate, Egypt. Subjects were recruited from home visits, geriatric clubs, and outpatient clinics. The Montreal Cognitive Assessment tools (MoCA & MoCA-B) were used to assess the prevalence of cognitive impairment, Hachinski ischemic score (HIS) to investigate the type of cognitive impairment, Ain Shams Cognitive Assessment (ASCA) tool to assess the pattern of specific cognitive domain affection, and an Egyptian socioeconomic status (SES) scale to classify the SES of the study participants.

**Results:**

The prevalence of cognitive impairment was 50.2% distributed as 37.7% for mild cognitive impairment (MCI) and 12.5% for dementia. The most common type of cognitive impairment was the degenerative type (47.9%). Pattern of specific domain affection among cognitively impaired subjects ranged from 94% for visuospatial function to 12.7% for abstraction. Cognitive impairment was significantly higher with increasing age, female sex, marital status (single or widow), low education, higher number of comorbidities, and positive family history of cognitive impairment (*p* < 0.001). Also, cognitive impairment was concentrated mainly among participants with low socioeconomic score (*p* < 0.001).

**Conclusion:**

In Egypt, cognitive impairment is significantly prevalent and concentrated among those who are in low socioeconomic status. Patients with mild CI were more than those with dementia, and the most common type of CI was the degenerative type. Increasing educational level of low SES population and improving their access to healthcare services are highly recommended to improve the inequity of cognitive impairment.

## Introduction

Among the world’s population, the elderly comprises a rapidly expanding demographic group, representing the fastest-growing segment [1]. Cognitive impairment (CI), as an age-related health problem, imposes a significant burden on families and caregivers. With the rapid aging of the population in recent years, there is an anticipation of a substantial rise in the prevalence of CI, making it a significant global public health concern [2].

The World Health Organization (WHO) estimates that the number of individuals with dementia worldwide is approximately 55 million, with this number expected to reach approximately 78 million by 2030 and 139 million by 2050 [3]. Two-thirds of the people with dementia are projected to be from the low- and middle-income countries including Africa [4]. Dementia prevalence in Africa varies from 2.3 to 20.0% [5], and a review on the epidemiology of dementia in the Middle East and North Africa (MENA) estimated a crude incidence of 27/1000 over a 20-year period [6]. In Egypt, a systematic review found the prevalence of dementia ranged from 2.1 to 5.7%; these data were derived from only four governments in Egypt (New Valley, Red Sea, Assiut, and Qena) [7].

Cognitive abilities serve as a fundamental cornerstone, enabling one to live independently, proficiently handle financial responsibilities, adhere to medication regimens, and drive safely. Moreover, the preservation of cognitive functions is vital for effective interpersonal communication, sensory information processing, integration, and appropriate responsiveness to others [8]. Under the broad umbrella of cognitive abilities, several distinct cognitive domains are present, each contributing to overall mental performance. These domains include attention, memory, executive function, language, and visuospatial abilities. With aging, each of these domains exhibits measurable decline [9].

Indeed, numerous risk factors have been elucidated in relation to the onset of cognitive impairment. These encompass a range of factors, including biological, lifestyle, environmental, and pathological influences that are linked to specific medical conditions and diseases [10]. These factors can influence cognition through life in both positive and negative ways. Factors that contribute positively include engaging in higher education, social interactions, seeking intellectual stimulation, and maintaining regular physical activity. Conversely, factors such as limited educational attainment, insufficient physical activity, health problems, and substance abuse (including alcohol and drug misuse) can yield detrimental effects on cognitive function [11].

The impact of social determinants on cognitive health is a universal and enduring phenomenon that operates persistently and cumulatively [12]. The dynamic nature of socioeconomic status (SES) extends through the entirety of an individual’s lifespan, wherein a variety of socioeconomic indicators exert distinct influences on cognitive well-being. These multifaceted indicators interact synergistically, contributing to the onset and progression of cognitive impairment. This highlights the critical role played by multiple individual socioeconomic risk factors in shaping cognitive health [13, 14].

In developing countries, disparities in SES have been observed to influence the diversity in cognitive performance and rates of cognitive decline [12]. Therefore, it is important to understand the contributing factors that result in varying levels of cognitive health within lower and higher socioeconomic groups among Egyptian populations. Furthermore, there is a gap regarding the epidemiological data on cognitive impairment in Egypt. Research on the prevalence of dementia should include multiple regions and investigate the underlying risk factors. Additionally, the extent and patterns of impairment across the range of cognitive domains are not yet well established among Egyptian populations. This study aimed to assess the prevalence of cognitive impairment and its sub-domains among community-dwelling elderly and to examine its relation to socioeconomic status. The findings from this research will provide population data evidence for researchers and policy makers to be used for informed decision-making.

## Methods

### Study design and settings

This is a cross-sectional study of 470 participants aged 60 years and older from community-dwelling elderly living in Kafr El-Sheikh Governorate in Egypt. The study participants were recruited from home visits and geriatric clubs and from patients attending outpatient clinics including geriatrics, ophthalmic, and physiotherapy clinics, using a convenient sampling method. The study was conducted during the period from 1 May 2020 to 1 October 2022. The data collection was carried out during the COVID-19 pandemic that resulted in long time of data collection due to limited access to the study participants.

### Sampling

The sample size was calculated based on the average estimate of prevalence of cognitive impairment among community-dwelling elderly in Egypt (5.7%) [7]. A sample size of at least 470 participants produces a two-sided 95% confidence interval. This sample was satisfactory to compare the SES mean score of the two groups by the two independent samples *t*-test with a moderate effect size of 0.5 at level of significance = 0.05 and power = 80%.

### Target population

The study included males and females’ community-dwelling elderly (> 60 years old) who were willing to participate in the study. Subjects with delirium, severe depression, acute illness, or communication disability that interfere with applying the assessment tools, e.g., severe hearing or visual impairment, were excluded.

### Data collection

The following assessments were done for each participant.

#### A structured interview questionnaire was designed to collect the following data

Including personal data such as age; sex; marital status; educational level (low educated is illiterate or ≤ 9 years of education and high educated > 9 years of education); smoking history; medical history of chronic diseases, e.g., diabetes mellitus and hypertension; and family history of cognitive impairment.

#### Cognitive function assessment

The Arabic version of the Montreal Cognitive Assessment (MoCA) tool was used [15]. It is a cognitive screening test that has been proven to be sensitive to mild cognitive impairment (MCI) and can predict future cognitive decline in several cognitively impaired states, including Alzheimer’s disease (AD). It is also useful in differentiating between MCI and AD in mild to moderate stages. It assesses different cognitive domains including attention and concentration, executive functions, memory, language, visuospatial skills, conceptual thinking, calculations, and orientation. The time for its application is 10–15 min, with a maximum score of 30. A score of 26 or more is considered normal, score of 19–25 is considered MCI, and score of 18 or less is considered dementia [16].

The author of the MoCA proposed adding 1 point to individuals with 12 years of education or less, aiming to correct the effect of education on MoCA performance and developed a specific version adapted for education-limited individuals named MoCA-Basic (MoCA-B) version [17]. We used (MoCA) version [16] for educated subjects and (MoCA-B) version [17] for illiterate and low-educated (< 12 years of education) subjects.

#####  For assessment of specific cognitive domain affection

Among cognitively impaired subjects, we used a new valid and reliable neurocognitive diagnostic evaluation battery that can evaluate specific domain affection in both educated and illiterate subjects under the name of Ain Shams Cognitive Assessment (ASCA) scale [18]. This scale includes the following cognitive subtests: paired associated test [verbal learning (VL), distractor interval (DI), delayed recall (DR)], word recognition test (WRNP), Bender-Gestalt (BG) copy and recall, digit span forward (FW) and backward, set shifting line and time of trail-making test, verbal fluency lexical and semantic, confrontation object naming (CN), cuing (stimulus or phonemic), abstraction, and judgment. These subtests can assess several cognitive domains including learning and verbal memory, working memory (encoding, spatial, cuing), language and semantic memory, executive function and processing speed, visuospatial function, attention, abstraction, and judgment. Each domain was assessed separately to see if it is normal or impaired. In this study, we defined test and domain impairment as z-scores falling below − 1.5, equivalent to at least 1.5 standard deviations (SDs) below the mean of the normative population. This criterion aligns with the midpoint of the range (1–2 SD) suggested in the DSM-5 as a reference for mild cognitive disorders [19].

#### Type of cognitive impairment

This was assessed using Hachinski ischemic score (HIS) which categorize CI into primary degenerative, vascular, or multi-infarct and mixed type. The composing items include history of hypertension and history of stroke as well as symptoms suggesting cerebrovascular events. The total score is determined as follows: < 4 suggests a degenerative type, score 4–7 for mixed type, and > 7 suggests a vascular type [20].

#### Depression assessment

This was performed using Arabic version of Patient Health Questionnaire-9 (PHQ-9) [21]. PHQ-9 is a clinical tool for assessment of depression. The participant is asked nine questions: “over the last 2 weeks, how often have you been bothered by any of those problems? For every question, participant is scored as follows: not at all = 0, several days = 1, more than half the days = 2, and nearly every day = 3. The total score for depression severity is classified as follows: 0–4 none or minimal depression*,* 5–9 mild depression, 10–14 mild to moderate depression, 15–19 moderate depression, and 20–27 severe depression. Participants with moderate or severe depression were excluded [22].

#### Socioeconomic status (SES)

This was calculated based on the Egyptian Socioeconomic Scale [23]. The scale includes several subdomains such as level of education, work status, computer use, income, family size, crowding index, sewage, and refuse disposal, each domain has a specific score, then the total score was calculated (maximum score = 48), and the cut-off points used for SES classification were as follows: high level was indicated as at least 70% (≥ 33), medium level as 40 to less than 70% (19–33), and low level as less than 40% (< 19).

The interview was conducted by a trained geriatrician and consumed on average about 50 min for each participant to be completed.

### Statistical analysis

The collected data underwent coding, tabulation, and statistical analysis using SPSS version 22 (IBM Corp., Armonk, NY, USA). Quantitative variables were described in terms of their mean, standard deviation (SD), and range. Qualitative variables were described by frequency and percentage. To compare qualitative variables, the chi-square test was employed, and in cases where there was an expected cell count of less than five, Fisher’s exact test was used. All statistical tests were two tailed, and the significance level was determined based on the probability (*P*) value, where *p* < 0.05 was deemed significant, and *p* < 0.01 was regarded as highly significant. The analysis of ASCA subtests impairment was done using Z-scores analysis, considering positive and negative deviations around the standardized Z score using the mean and SD of the normative Egyptian populations.

## Results

**Fig. 1:**
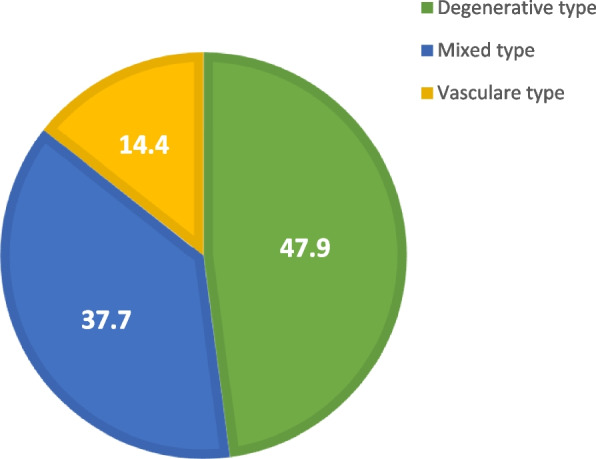
Types of cognitive impairment assessed by Hachinski ischemic score among cognitively impaired participants (*n* = 236)

A total of 470 subjects were investigated in this study; the mean age of the study population was 66.3 years old with standard deviation ± 5.6. Most of them were males (72.1%), married (67.9%), low educated (58.5%), and nonsmoker (52.8%) and have medium level of SES (65.1%) (Table 1). The prevalence of cognitive impairment (CI) according to MoCA test examination was 50.2% which was distributed as 37.7% for MCI and 12.5% for dementia (Table 2). By investigating the type of cognitive impairment using HIS, it was found that degenerative type was the most common (47.9%) followed by mixed type (37.7%) and then vascular type (14.4%) (Fig. 1).

**Table 1:** Sociodemographic data of the studied Egyptian elderly, 2020–2022 (*n* = 470)

Age (in years)	Range	60–90	
	Mean ± SD	66.3 ± 5.6	
		No.	%
**Age (years)**			
60–69		359	76.4
70–79		91	19.4
≥ 80		20	4.2
**Sex**			
Male		339	72.1
Female		131	27.9
**Marital status**			
Married		319	67.9
Single		14	2.9
Widow		133	28.3
Divorced		4	0.9
**Education**			
Low education (≤ 9 years)		275	58.5
High education (> 9 years)		195	41.5
**Smoking**			
Nonsmoker		248	52.8
Smoker		157	33.4
Ex-smoker		65	13.8
**SES level**			
High		86	18.3
Medium		306	65.1
Low		78	16.6

*SES* Socioeconomic status

**Table 2:** Prevalence of cognitive impairment assessed by Montreal Cognitive Assessment (MoCA) test among studied Egyptian elderly, 2020–2022 (*n* = 470)

MoCA test		
	**No.**	**%**
**Normal**	234	49.8
**Cognitive impairment**		
MCI	177	37.7
Demented	59	12.5
**Range of MoCA test score**	3–30	
**Mean ± SD**	22.8 ± 5.5	

*MoCA* Montreal Cognitive Assessment, *MCI* mild cognitive impairment

The prevalence of CI exhibited a notable degree of inequality. The results showed that older age, female gender, being unmarried (single, widow, divorced), and having a lower educational level were significantly associated with higher rates of CI. Additionally, CI was significantly associated with increasing total number of comorbidities (≥ 3) (*p* < 0.001) with a higher prevalence observed in individuals with chronic medical conditions. CI was significantly high among older adults with diabetes 64.2% (46% MCI and 18.2% dementia), hypertension 56.1% (36.9% MCI and 19.2% dementia), neurological disorders 76.8% (42% MCI and 34.8% dementia), and sensory impairment 69.3% (41.9% MCI and 27.4% dementia). Furthermore, CI was significantly associated with positive family history of cognitive impairment (*p* = 0.002) such that CI was significantly higher among subjects with positive family history (70.8%) compared to (47.9%) among subjects with no family history of CI (Table 3).

**Table 3:** Association between cognitive function (normal, MCI or dementia) and sociodemographic and medical data of the studied Egyptian elderly, 2020–2022 (*n* = 470)

Variable	MoCA test						*p*-value
	**Normal (*n* = 234)**		**MCI (*n* = 177)**		**Dementia (*n* = 59)**		
	**No.**	**%**	**No.**	**%**	**No.**	**%**	
**Age (years)**							< 0.001^*^
60–69	214	59.6	131	36.5	14	3.9	
70–79	20	21.9	41	45.1	30	33.0	
≥ 80	0	0	5	25.0	15	75.0	
**Sex**							0.047^*^
Male	178	52.5	116	34.2	45	13.3	
Female	56	42.7	61	46.6	14	10.7	
**Marital status**							< 0.001^*^
Married	200	62.7	102	32.0	17	5.3	
Single	6	42.9	7	50.0	1	7.1	
Widow	26	19.6	66	49.6	41	30.8	
Divorced	2	50.0	2	50.0	0	0.0	
**Education**							< 0.001^*^
Low education	123	44.7	115	41.8	37	13.5	
High education	111	56.9	62	31.8	22	11.3	
**Smoking**							< 0.001^*^
Nonsmoker	133	53.6	94	37.9	21	8.5	
Smoker	86	54.8	54	34.4	17	10.8	
Ex-smoker	15	23.1	29	44.6	21	32.3	
**Number of comorbidities**							< 0.001^*^
No comorbidities	38	82.6	6	13.0	2	4.4	
1–2 comorbidities	137	64.6	69	32.5	6	2.9	
≥ 3 comorbidities	59	27.8	102	48.1	51	24.1	
**Types of comorbidities**							< 0.001^*^
DM							
**Yes**	63	35.8	81	46.0	32	18.2	
**No**	171	58.2	96	32.6	27	9.2	
Hypertension							
**Yes**	87	43.9	73	36.9	38	19.2	< 0.001^*^
**No**	147	54.0	104	38.2	21	7.8	
Neurological disorders^#^							
**Yes**	16	23.2	29	42.0	24	34.8	< 0.001^*^
**No**	218	54.4	148	39.9	35	8.7	
Sensory impairment^#^							
**Yes**	38	30.7	52	41.9	34	27.4	< 0.001^*^
**No**	196	56.6	125	36.2	25	7.2	
**Family history of cognitive impairment**							0.002^*^
**Yes**	14	29.2	22	45.8	12	25.0	
**No**	220	52.1	155	36.7	47	11.2	

^*^Significant. ^#^Neurological disorders such as cerebrovascular stroke, Parkinson disease, epilepsy, and brain tumors. ^#^Sensory impairment such as visual or hearing impairment

There was a statistically significant association between cognitive function and all socio-economic domains except for family size and crowding index as cognitive impairment was significantly associated with low education, no occupation, low income, infrequent computer uses, and bad sanitation (*p* < 0.001). The prevalence of CI was mainly concentrated in subjects with low and medium SES. Dementia rate was significantly higher among subjects with low SES compared to those with high SES (25.6% vs. 4.6%). Similarly, MCI rate was significantly higher among subjects with low SES compared to those with high SES (60.3% vs. 22.1%) with significant difference (*p* < 0.001) (Table 4).

**Table 4:** Association between cognitive function and different socio-economic (SE) domains of the SES scale among the studied Egyptian elderly, 2020–2022 (*n* = 470)

Socio-economic domains	MoCA test							
	**Normal (*n* = 234)**		**MCI (*n* = 177)**		**Dementia (*n* = 59)**			
	**No.**	**%**	**No.**	**%**	**No.**	**%**	***χ***^**2**^	***p*-value**
**Mother education**							42.326	< 0.001^*^
Illiterate	103	38.9	123	46.4	39	14.7		
Primary	62	57.9	28	26.2	17	15.9		
Preparatory	33	61.1	18	33.3	3	5.6		
Secondary	32	80.0	8	20.0	0	0.0		
University	4	100.0	0	0.0	0	0.0		
**Father education**							59.194	< 0.001^*^
Illiterate	29	28.2	53	51.4	21	20.4		
Literate certificate	26	47.3	20	36.3	9	16.4		
Primary	49	62.0	26	32.9	4	5.1		
Preparatory	38	38.0	45	45.0	17	17.0		
Secondary	70	64.2	31	28.5	8	7.3		
University	21	91.3	2	8.7	0	0.0		
Postgraduate	1	100.0	0	0.0	0	0.0		
**Mother work**							27.473	< 0.001^*^
No	180	45.0	161	40.2	59	14.8		
Yes	54	77.1	16	22.9	0	0.0		
**Father work**							29.799	< 0.001^*^
No	11	20.4	26	48.1	17	31.5		
Yes	223	53.6	151	36.3	42	10.1		
**Computer use**							115.985	< 0.001^*^
Never	77	28.7	138	51.5	53	19.8		
Sometimes	144	76.2	39	20.6	6	3.2		
Lot of time	13	100.0	0	0.0	0	0.0		
**Per capita income**							55.363	< 0.001^*^
Loan not repaid	0	0.0	1	100.0	0	0.0		
Big loan	5	35.7	6	42.9	3	21.4		
Small loan	28	27.7	59	58.4	14	13.9		
Enough only	94	45.9	81	39.5	30	14.6		
Enough and saving	107	71.8	30	20.1	12	8.1		
**Family size**							7.880	0.096
6	50	43.5	45	39.1	20	17.4		
5	105	48.8	81	37.7	29	13.5		
< 5	79	56.4	51	36.5	10	7.1		
**Crowding index**^a^							3.369	0.185
2–4	134	46.9	111	38.8	41	14.3		
< 2	100	54.3	66	35.9	18	9.8		
**Sewage disposal**							12.857	0.002^*^
No	3	13.6	13	59.1	6	27.3		
Yes	231	51.6	164	36.6	53	11.8		
**Refuse disposal**							42.489	< 0.001^*^
No	45	28.7	86	54.8	26	16.5		
Yes	189	60.4	91	29.1	33	10.5		
**SES level**							61.338	< 0.001^*^
High	63	73.3	19	22.1	4	4.6		
Medium	160	52.3	111	36.3	35	11.4		
Low	11	14.1	47	60.3	20	25.6		

^*^Significant

^a^The total number of residents per household divided by the number of bedrooms available in the home excluding kitchen, bathroom, and balconies

**Fig. 2:**
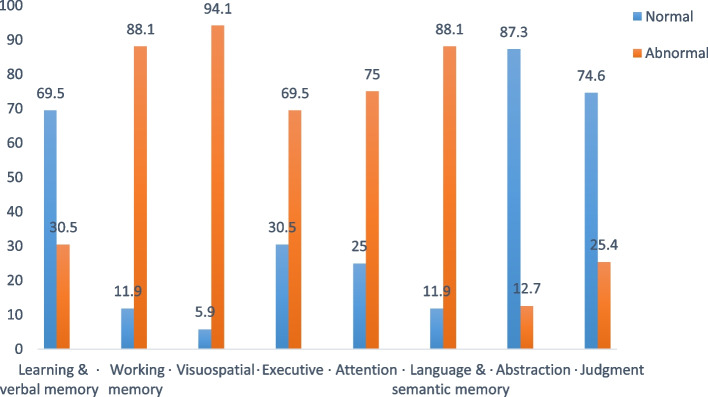
Analysis of cognitive domains function among CI studied Egyptian elderly, 2020–2022 by ASCA scale (*n* = 236). ASCA, Ain Shams Cognitive Assessmen scale. Normal, normal cognitive testing by ASCA. Abnormal, impaired cognitive testing by ASCA

By examining the pattern of distribution of specific cognitive domain affection among cognitively impaired subjects using ASCA scale, the most affected domain was visuospatial function (94.1%) followed by language and semantic memory (88.1%) and working memory (88.1%), and the least was for abstraction function (12.7%) (Fig. 2).

When comparing the cognitive performance across cognitive domains and the educational level, it was found that among the highly educated group, there was a significant higher affection of working memory function, executive functions, and learning and verbal memory than in low educated group (100% vs. 81.5%, *p* < 0.001), (83.3% vs. 61.8%, *p* < 0.001), and (48.8% vs. 20.3%, *p* < 0.001), respectively, while among low educated group there was a significant higher affection of the visuospatial function and language and semantic memory functions than in the high educated group (99.3% vs. 84.5%, *p* < 0.001) and (93.4% vs. 78.6%, *p* < 0.001). However, there was no significant difference between the two groups as regard performance in the cognitive functions of attention, abstraction, and judgment (*p* = 0.209, 0.343, and 0.179, respectively) (Table 5).

**Table 5:** Relationship between cognitive domain impairment and the educational level (high and low) among studied Egyptian elderly with cognitive impairement, 2020–2022 (*n* = 236)

Cognitive domain	Education level					
	**Low education**		**High education**			
	*N* (152)	%	*N* (84)	%	*Χ*^2^	*p*-value
**Learning and verbal memory**					20.604	< 0.001^*^
Normal	121	79.6	43	51.2		
Impaired	31	20.4	41	48.8		
**Working memory**					17.557	< 0.001^*^
Normal	28	18.4	0	0.0		
Impaired	124	81.6	84	100.0		
**Visuospatial**					21.289	< 0.001^*^
Normal	1	0.7	13	15.5		
Impaired	151	99.3	71	84.5		
**Executive**					11.786	0.001^*^
Normal	58	38.2	14	16.7		
Impaired	94	61.8	70	83.3		
**Attention**					1.577	0.209
Normal	42	27.6	17	20.2		
Impaired	110	72.4	67	79.8		
**Language & semantic memory**					11.409	0.001^*^
Normal	10	6.6	18	21.4		
Impaired	142	93.4	66	78.6		
**Abstraction**					0.898	0.343
Normal	135	88.8	71	84.5		
Impaired	17	11.2	13	15.5		
**Judgment**					0.179	0.672
Normal	112	73.7	64	76.2		
Impaired	40	26.3	20	23.8		

^*^Significant

When comparing cognitive subdomain impairment by ASCA and SES level (high, low, medium), we found that there was significant association between SES level and impairment in the following cognitive sub-domains: episodic verbal recall, processing speed, language and semantic memory, and visuospatial function. That impairment in those domains was higher among low SES group compared with high SES group (37.3% vs.13.1%, *p* = 0.037) for verbal recall, (26.9% vs.4.3%, *p* = 0.007) for processing speed, (95.5% vs. 69.6%, *p* = 0.004) for language and semantic memory, and (91.1% vs. 60.9%, *p* < 0.001) for visuospatial function (Table 6).

**Table 6:** Relationship between impairment in cognitive sub-domain by ASCA scale and SES level of CI studied Egyptian elderly, 2020–2022 (*n* = 236)

Impairment	**SES level**						***p*-value**
	**High (*n* = 23)**		**Medium (*n* = 146)**		**Low (*n* = 67)**		
	**No.**	**%**	**No.**	**%**	**No.**	**%**	
**Learning & verbal memory**							
Verbal learning	4	17.4	26	17.8	19	28.4	0.194
Verbal recall	3	13.1	35	23.9	25	37.3	0.037^*^
**Working memory**							
Working encoding	18	78.3	113	77.4	58	86.6	0.290
Working spatial	20	86.9	136	93.2	66	98.5	0.097
Working cuing	8	34.8	41	28.1	23	34.3	0.587
**Executive functions**							
Executive function	19	82.6	92	63.0	50	74.6	0.071
Processing speed	1	4.3	18	12.3	18	26.9	0.007^*^
**Attention**	16	69.6	111	76.1	50	74.6	0.799
**Language & semantic memory**	16	69.6	128	87.7	64	95.5	0.004^*^
**Visuospatial**	14	60.9	94	64.4	61	91.1	< 0.001^*^
**Abstraction**	4	17.4	16	10.9	10	14.9	0.562
**Judgment**	3	13.1	36	24.7	21	31.3	0.208

*ASCA*, Ain Shams Cognitive Assessmen scale, *SES*, socioeconomic status

^*^Significant

## Discussion

With the increasing proportion of elderly individuals within the population, CI emerges as a significant public health concern, posing threats to the independence of older adults and exerting profound challenges on the social security and healthcare systems [1]. According to MoCA test examination of the studied population, the estimated prevalence of CI in this study was 50.2% with 37.7% who had MCI, and 12.5% were demented. This figure is consistent with an Egyptian study that estimated the prevalence of CI in community-dwelling elderly as 51.4% [24]. The prevalence of CI worldwide varies widely. In a systematic review (including 80 studies), the estimated prevalence of CI ranged between 5.1 and 41% with a median of 19.0% [25]. These variations can be attributed to various factors including the study settings, demographic characteristics of the population, cultural differences, and variations in the assessment tools used for screening. These factors collectively contribute to variations in the definition and categorization of mental and neurocognitive disorders, thereby influencing the wide range of CI prevalence reported [26].

The prevalence of MCI in Egypt was estimated as 32% by Amer et al. [27]. Another study also found the prevalence to be 34.2 and 44.3% of the elderly men and women, respectively [15]. That was consistent with the result of the current study that the prevalence of MCI was 37.7%. The higher prevalence of CI, both MCI and dementia, within the Egyptian population can potentially be attributed to the significant proportion of illiteracy among Egyptian elderly individuals, accounting for approximately 56.5% [28]. In the present study, illiterate individuals or those with lower educational levels represented 58.5% of the studied population that further emphasizes the association between educational attainment and cognitive health.

By investigation of the type of CI using the Hachinski ischemic score, in the present study, the degenerative type was the most common type among cognitively impaired subjects followed by mixed type then the vascular type. This is consistent with the classification of dementia in most literatures where the most common type of neurodegenerative dementia was Alzheimer and then vascular dementia [29, 30].

The extent and patterns of impairment across the range of cognitive domains are not yet well established so we used a newly developed validated tool named Ain Shams Cognitive Assessment (ASCA) tool that assesses specific cognitive domain functions among cognitively impaired subjects [18]. For easier comparison with other literature, we classified the cognitive domains into the most common domains classified by DSM-5 (learning and memory, complex attention, executive function, language, perceptual motor function) [19]. We found that the highest proportion of test impairment was for the figure copy and recall of Bender-Gestalt test (94%), naming (83%), and word recognition test (80%) which primarily assess visuospatial and memory functions respectively. These results suggest that patients were particularly impaired in visuospatial and memory domains. This is consistent with the type of cognitive impairment tested above where the most common type of impairment was the degenerative type. This is due to the common predominance of memory impairments in neurodegenerative-related cognitive impairment, compared with predominance of attention/executive in vascular-related impairment [31].

By examination of the distribution of patterns of impairment among CI participants, it was very diverse, and most of them were impaired on at least one cognitive domain with a very few participants who had cognitive performance at or above average expectations. This is consistent with previous study [32]. Patterns of impairment across cognitive domains were as follows: 94.1% for visuospatial function, 88.1% for working memory and for language and semantic memory functions,75% for attention, 69.5% for executive functions, 30% for learning and verbal memory, 25.5% for judgment, and 12.7% for abstraction. In another study, the prevalence of specific domain affection was as follows: 31.5% for visuospatial function, 41.2% for language, 41.7% for executive function, 42.2% for learning and memory, and 48.8% for complex attention [32]. Those lower prevalence rates than the current study may be attributed to the different data presentation method as they assessed the pattern of domain affection in the total study population, while in the current study, we assessed it among the cognitively impaired subjects only. Therefore, the proportion of impairment was higher in the current study. Furthermore, the distribution of the pattern was different, which could be attributed to different risk factors. Their study was conducted among hemodialysis patients who have vascular risk factors for vascular dementia which was reflected on higher affection of attention and executive functions.

The diversity in cognitive performance and varying rates of cognitive decline have been documented to undergo alterations in relation to a range of factors, including demographic characteristics, educational background, lifestyle choices, physical well-being, social engagement, and economic resources [33]. Testing the relationship between cognitive function and socioeconomic domains in the current study revealed that CI (MCI or dementia) was significantly associated with low educational level, unemployment, low income, limited computer use, and bad sanitary condition. In the same context, a recent study revealed that older adults who reported lower perceived income, lower educational attainment, compromised physical and mental health, and limited access to physical and social resources were found to have a higher likelihood of CI [34]. Moreover, it is important to note that the influence of these sociodemographic characteristics on cognitive function is not uniform, as they can interact with one another, giving rise to unique patterns of cognitive performance [35].

By examination, in the effect of SES level on performance across different cognitive domains, we found that there was significant association between SES level and impairment in the following cognitive sub-domains (episodic verbal memory, language and semantic memory, processing speed, and visuospatial functions). Impairment of those sub-domains was higher in low SES subjects in comparison with high and medium SES subjects with significant difference. Although impairment in the working spatial function was the most common sub-domain impairment among the three groups, there was no statistically significant difference between them. The observed distribution of cognitive domain impairment can be explained by exposure to persistent chronic stressors that have been linked to reductions in hippocampal and amygdala volume, as well as atypical activity in the prefrontal cortex. These brain regions play a vital role in various cognitive functions, including memory, emotion processing, executive functions, and social behavior [36].

A substantial body of literature demonstrates a consistent and independent association between socioeconomic status and cognitive function in later stages of life [33–35]. This was confirmed in the present study where there was a highly significant association between cognitive function and SES level. The mechanism by which SES impacts cognitive impairment is thought to be through the building and preservation of brain reserve capacity [35]. The concept of cognitive reserve highlights the brain’s remarkable capacity for flexibility and adaptability, enabling it to actively counteract the impact of age- or disease-related alterations within its networks [37].

It is widely accepted that low SES is one of the risk factors for CI in older adults. Individuals with lower SES often have limited health literacy due to their lower levels of education. Additionally, they are less likely to receive health advice and have reduced motivation to undergo CI screening, which is compounded by limited access to health resources. This economic disparity also results in reduced social participation, as low SES individuals may lack the time and energy to engage in socially enriching activities that can expand their cognitive reserve and buffer the risk of CI. Thus, low SES populations are more susceptible to CI [38].

In contrast, individuals from higher socioeconomic groups are typically more advantaged with regards to health. Their good working and living conditions and greater access to healthcare knowledge and medical technology, resulting from their educational background, occupational status, and income, make them less susceptible to health injuries and better able to prevent cognitive decline. Furthermore, they are more inclined towards a healthy lifestyle and social network, which can help delay cognitive decline [39]. Even when cognitive decline occurs, those with higher SES have a better chance of detecting the condition early and correcting adverse factors to avoid further deterioration of cognitive function [40]. Therefore, there is an urgent need to prioritize efforts aimed at enhancing cognitive function and preventing the progression from MCI to dementia, particularly among older adults who are at higher risk, including those from low SES backgrounds. Ensuring improved access to healthcare services becomes a critical focus in addressing the needs of this vulnerable population.

### Limitations

This study has some limitations with generalizability as the sample size was a convenient sample, and also, the design was a cross-sectional study that could not assess the actual causal effect of different socioeconomic indicators (education, work stat, computer use, income, etc.) and impairment in specific cognitive domain. So, more longitudinal studies with larger sample sizes focusing on investigating the underlying risk factors for CI and its inequity among Egyptian elderly in various regions in Egypt are needed.

## Conclusion

This study found that cognitive impairment among community-dwelling elderly in Egypt was prevalent, and the most common type of CI was the degenerative type. There was impairment in at least one cognitive domain, and co-occurrence of impairment across domains was very common. The most affected cognitive domain was the visuospatial function, and the least affected one was abstraction. The working memory function was the most affected domain among the highly educated group, while among illiterate and low educated group the visuospatial function was the most affected domain. CI was significantly associated with increasing age, female gender, multiple comorbid conditions, and positive family history of CI. It also had a highly significant association with SES level that it was mainly concentrated in the socioeconomically disadvantaged population (low educational level, non-occupied, limited computer use, low income, and bad sanitation).

A significant association was observed between SES level and impairment in the following cognitive sub-domains: episodic verbal memory, language and semantic memory, processing speed, and visuospatial functions. Enhancing the educational level of low SES population and improving their access to healthcare services can contribute to reducing the disparities in cognitive impairment.

## Data Availability

The datasets used and/or analysed during the current study are available from the corresponding author on reasonable request.

## Abbreviations

AD: Alzheimer’s disease
ASCA: Ain Shams Cognitive Assessment
BG: Bender Gestalt
BW: Backward
CI: Cognitive impairment
CN: Confrontation naming
DI: Distractor interval
DR: Delayed recall
FW: Forward
HIS: Hachinski ischemic score
MCI: Mild cognitive impairment
MoCA: Montreal Cognitive Assessment
SD: Standard deviation
SES: Socioeconomic status
VL: Verbal learning
WRNP: Word recognition novel prototype
